# Development of personalized profiles of students with autism spectrum disorder for interactive interventions with robots to enhance language and social skills

**DOI:** 10.3389/fpsyt.2024.1455627

**Published:** 2024-11-13

**Authors:** Javier Herrero-Martín, David Fonseca, Selene Caro-Via, Xavi Canaleta

**Affiliations:** ^1^ Department of Preprimary and Primary Education. Education Faculty of La Salle University Center, Universidad Autónoma de Madrid, Madrid, Spain; ^2^ Technology Enhanced Learning line of the Human Environment Research Group, La Salle, Ramon Llull University, Barcelona, Spain

**Keywords:** educational robotics, social robotics, educational innovation, inclusion, diversity, autism spectrum disorder

## Abstract

The inclusion of students with autism spectrum disorder (ASD) in mainstream education (primary and secondary, in the range of 4-5 to 8-10 years old) is a complex task that has long challenged both educators and health professionals. However, the correct use of digital technologies such as personalization settings and interaction with robots has clearly shown how these new technologies can benefit ASD students. However, it is essential to characterize the profile, problems, and needs of each student, since it is not possible to generalize an accessible approach for all users. The work presented shows the creation and validation, through pilot tests, of an instrument that outlines the main needs of a student with ASD, based on behavioral variables. In a later phase, instructional sequences will be designed and adapted through digital tablets and interaction with a robot to improve specific aspects identified in the initial profile. The results demonstrate the method’s ability to assess and prioritize profiles satisfactorily which helps create a design adjusted to each student. The first pilot tests have been well received by ASD students, who have shown increased interest in the contents and methods used in this approach. Motivation levels and engagement have also increased, and social interactions with their peers have improved.

## 1 Introduction

Autism is a neurodevelopmental disorder currently affecting 0.6% of the global population (1). It is generally characterized by the altered establishment and management of social relationships and restrictive or repetitive behavior patterns, interests, or activities (Updates to *DSMV-TR Criteria Text*, and *ICD-11 Codes* 2024). The disorder manifests itself in diverse forms which include motor, verbal, cognitive-intellectual, social, and emotional behavior deficits or alterations (2). Despite notable efforts to improve the categorization of the disorder, further research is needed to achieve a better alignment of evaluative and diagnostic procedures to help ensure that future developments attain greater reliability and facilitate the generalization of results to similar patterns (3).

The complex nature of autism spectrum disorder (ASD) requires a thoughtful reflection on the diverse forms of intervention, from clinical, social, and educational viewpoints, which can involve various methods and tools to meet individual treatment goals (4). The combination of these three diagnostic determination variables suggests the need to personalize diagnostic practice beyond standard procedures, integrating findings from neuroscientific and methodological research with those derived inductively from evidence-based practice (5). This reality is particularly evident in the educational context, where the means-end adequacy for intervention must effectively combine both the rational disposition of technical and human resources (6) and the appropriate distribution of time and space to optimally achieve intervention objectives (7, 8).

Additionally, the need for a more personalized approach reflects the varying trajectories that autistic children experience during their early developmental years (9). Along with temporal analysis in diagnostic performance and review of the complex conceptual framework, the determination of individual status often faces variable interpretations by different agents involved in the detection and assessment process (10). In particular, interventions focusing on social interaction in ASD emphasize areas such as social cognition, peer relationships, and mutual and shared attention (11). Language and communication difficulties, present in varying degrees in 40% to 70% of the population, include developmental linguistic delays, stereotyped language use, and echolalia. Overall, social and communicative-linguistic impairments form a core focus of ASD evaluation and intervention (12). Given that social skills are considered the most stable predictor of success and social and educational well-being, their enhancement is crucial in any intervention plan (13).

Overall, the optimal matching of means and ends in ASD interventions must be linked to leveraging the technological benefits of individualized treatments, given the differences in key factors such as verbal and non-verbal communication, concentration difficulties, limited contextual interest, or limited adaptability to new situations (14). In this context, an initial evaluation is a critical point in the intervention process, as it establishes baseline conditions for promoting change, allowing for effective and scientifically interpretable transformation (15).

Among the array of options, social robotics is an emerging field in the psychoeducational treatment of ASD communication and language (16), including humanoid versions (e.g., NAO, Qtrobot, Isobot) and “animaloid” appearances (e.g., Pleo, Zoomer, POL) as well as other types like the robotic arm (17) or Pekoppa, the plant robot used in interpersonal synchronization research (18). Among these, exploring the possibilities of linking robotics with autism has largely utilized the NAO robot to enhance linguistic-communicative skills (19, 20). Particularly noteworthy are combined experiences where the robot is used alongside augmentative mobile devices to enhance the educational experience for children with ASD. However, despite current usability limitations (e.g., battery life, dependency on programming models), the combined use of robots, psycho-pedagogical design, and other means, allows for the social use of technology as a proven benefit of such interventions (21).

Previous studies have highlighted the importance of evaluating demographic profiles for the identification of customizable behavioral and emotional patterns in children with ASD, which has been a significant aid in the development of automated protocols based on supervised machine learning (ML) (22). These studies aim to facilitate specific interventions in ASD using robots, considering the diversity of cognitive-emotional conditions of the recipients (levels of valence, arousal, and engagement). Moreover, this type of intervention is proving to be fairly effective in comparison with human-mediated interventions [so-called Wizard of Oz scenarios (23)]. More recently, Konishi et al. (24) have demonstrated how the use of self-administered questionnaires, which allow for the discrimination of individual-associated characteristics, can add value to robotic interventions with children with ASD. These studies focused on the effectiveness of the supervised system in identifying and adapting the robot’s behavior to the individual conditions of the participants. However, the robot’s behavioral approaches to the subject’s emotional states (through recognition and social adjustment of emotional patterns) are only one part of the set of variables to consider. Social interaction (non-verbal communicative acts, communicative implications, linguistic uses, and manifestations) requires the combined coordination of multiple sources of information, not all of which are readily accessible. This mental competence shows significant differences even among autistic children with equivalent diagnostic patterns, further highlighting the need to refine the intervention profile beforehand to make accurate estimations of the effectiveness of robot-assisted treatments.

This study establishes a framework for an initial personalization to determine the optimal pattern for the use of the social robot NAO in autism spectrum disorder (ASD) interventions. Its purpose is driven by the need to select the baseline level of the subject to design a trial that allows for the assessment of various interventions, given that the facilitation of communicative and linguistic processes depends on a) the individual profile of the candidate; b) the characteristics of the intervention; and c) the establishment of a specific intervention baseline (communicative-linguistic competence). The effects of interventions can be contrasted over time concerning the individual’s behavioral, physiological, motor, and social response conditions, as well as to modifications in the intervention and objectives established in the intervention program. The integration of a module focused on the personalization of intervention sequence programming into the evaluation process will allow for a better alignment of the work objectives for a specific user (user-adapted robot programming, hereafter UARP).

## 2 Methodology

This proposal is part of the DivInTech research project (Use of Robotics to Enhance Instrumental Skills of Students with Autism Spectrum Disorder through the Development of New Inclusive Contexts, PID2022-140284OB-I00). This research and development project is supported by the Spanish State Program for the Promotion of Scientific-Technical Research and its Transfer, under the Ministry of Science, Innovation, and Universities (2023 call). Its goal is to generate knowledge about the use of social robotics in the instrumental development of communicative and linguistic skills in children with ASD.

In collaboration with educational centers that have a space for diversity attention and/or students with ASD, the objectives are set to identify profiles, classify them, design, and implement intervention sequences with feedback on their effectiveness to schools and families, and map school initiatives to improve the inclusion of ASD profiles in the educational environment.

To define the working method, it is necessary to consider that the clinical manifestations of the autism spectrum present a multivariate picture (25). Currently, the autism spectrum combines previously distinct conditions, such as autistic disorder, Asperger’s disorder, childhood disintegrative disorder, or pervasive developmental disorder (26). This leads to the coexistence of a wide diversity of behavioral patterns, in which the restricted behavior repertoire expresses high variability concerning socio-communicative, motor, and environmental dimensions (27).

Since basic research within the DivInTech program aims to develop communicative and linguistic competence in autistic children with established oral competence (expressive, communicative, and functional comprehensive use), the first step in the work process was to determine the functional profiles for intervention. Thus, the approach and control of the behavioral pattern, in the communicative-verbal and motor-behavioral planes, will allow for the appropriate comparative analysis approaches both among study subjects and compared to norm typical patterns (non-ASD subjects). The qualitative process followed is detailed in the following sections, indicating the process of identification/selection of students and their prioritization for the technological implementation process, which is beyond the scope of the current proposal.

### 2.1 Participants

The study sample consisted of 11 primary and secondary students from La Salle La Seu d’Urgell School. This sample is predetermined by those students with a prior diagnosis who have validated access to the ISIE (an acronym that defines the classroom for Intensive Support of Inclusive Education). Currently, in the regional context of Catalonia where the school is located, only those students with disorders that have been evaluated and diagnosed by the psycho-educational service of the Ministry of Education of the Generalitat de Catalunya are entitled to care follow-up and access to the resources and spaces designed for such purposes. For the academic year 2023-2024, 11 students aged between 5 and 13 had their condition and access to the SIEI of the study center validated.

The school selection criteria are based on two principles: having a program that addresses the diversity of students with ASD, allowing for the personalization of learning exercises, monitoring, and support, and having a dedicated space where these students can interact with their teachers and peers, engage in personalized activities, and where future interactions with robots can take place. If a school meets these two premises, it can proceed to the next steps in categorizing the students.

To support these 11 students, the center has three full-time educators/psychologists and 2 additional part-time support staff. These five individuals are well-acquainted with the ISIE students and are responsible for conducting the initial assessment that will determine the degree of ASD of the students. The procedure described in this study adds a critical element when establishing inclusion and exclusion criteria (see section 2.3, Procedure), as it allowed for the identification and prioritization of those users within the sample who were most suitable for the robot intervention. This was based on: a) higher social competence; b) adequate linguistic ability to interact dialogically with the robot and educators; and c) a limited repertoire of restrictive and repetitive motor behaviors. The result, as described below, is a hierarchically ordered list of users for the design and intervention of UARP programs.

### 2.2 Instruments, procedure, and data collection

**Figure 1:**
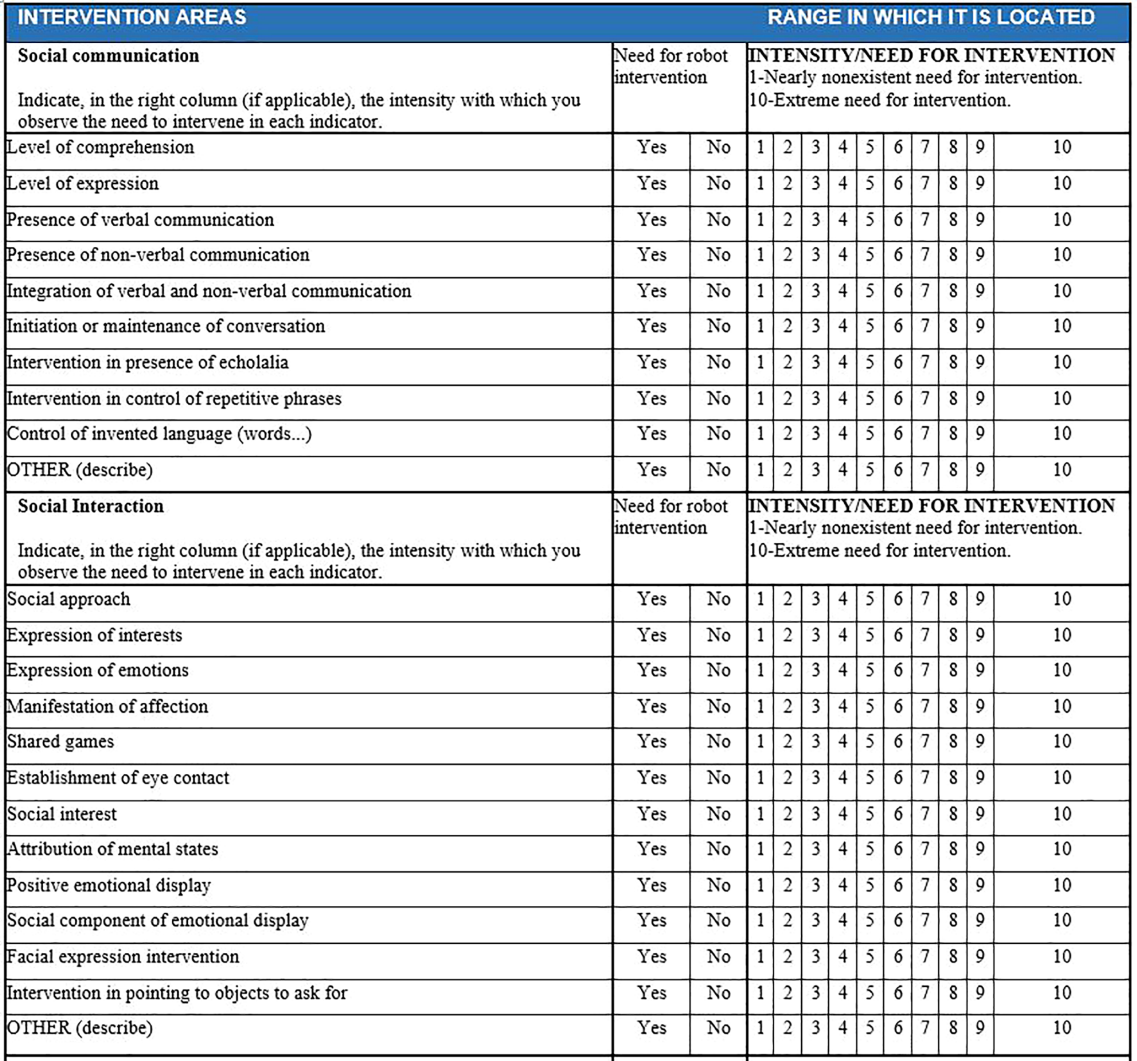
Dimensions of communication and social interaction.

**Figure 2:**
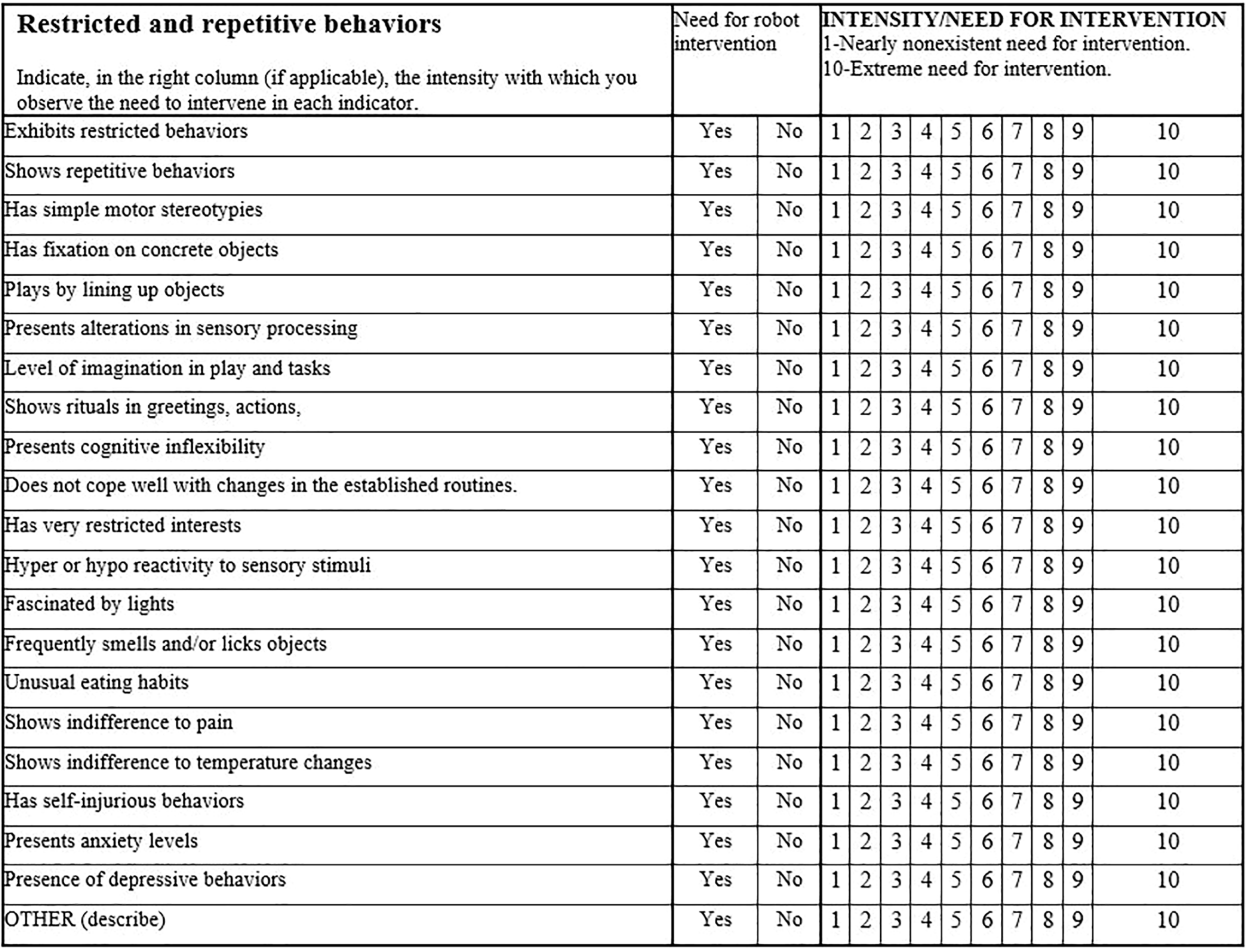
Dimensions of restricted and repetitive behaviors.

As an initial step, a behavioral observation register was created to determine profiles, based on the structure of indicators and diagnostic criteria from the DSMV-TR manual. **Figures 1**, **2** display the basic structure of the observational register, which includes 10 items for the social communication dimension, 12 items for social interaction, and 20 items for restricted and repetitive behaviors.

**Figure 3:**
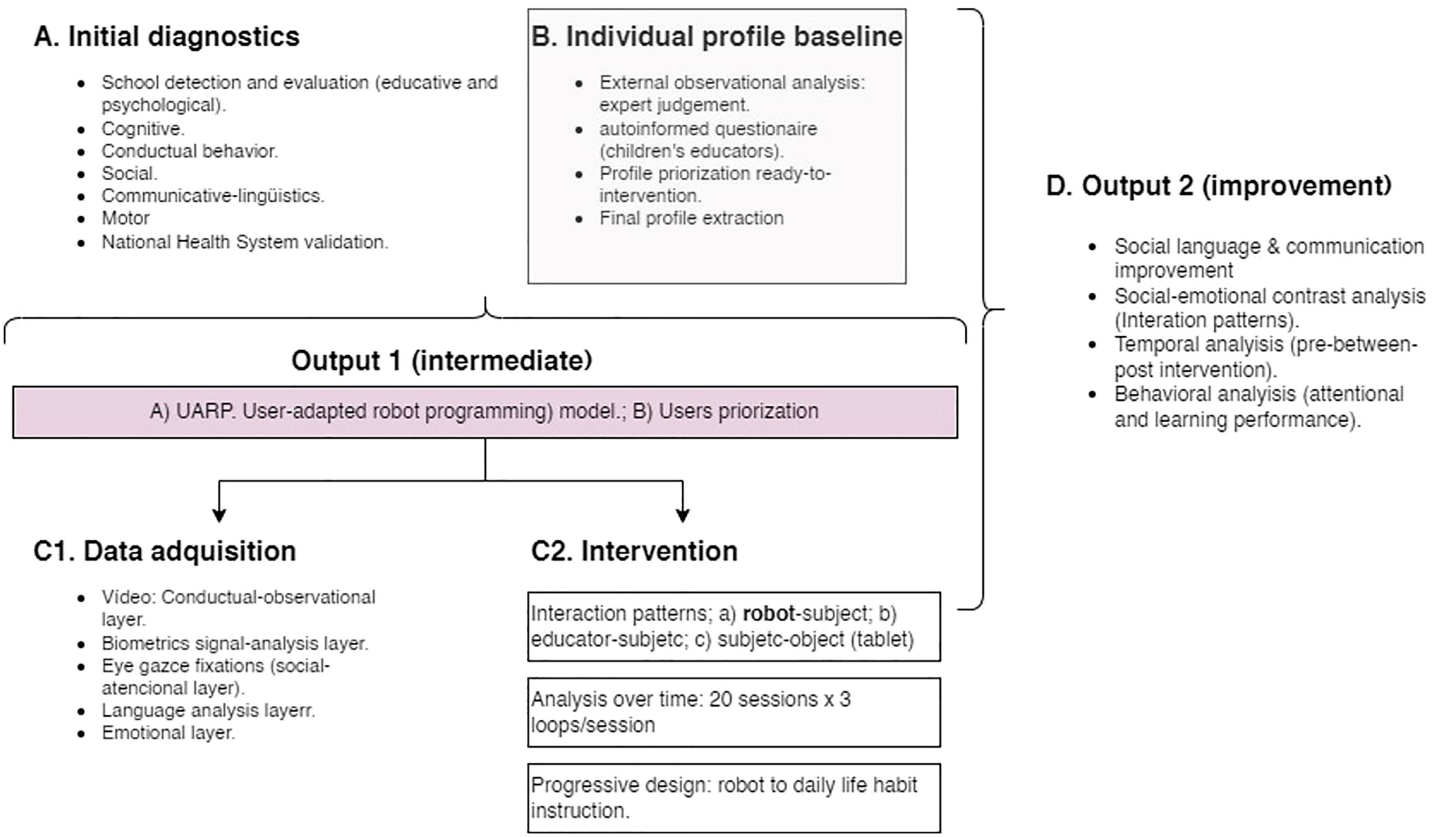
General research design. Shaded boxes (B and Out 1) show the UARP module and the intermediate output.

The overall design of the intervention, as shown in **Figure 3**, consists of four well-defined phases. The first phase establishes the diagnostic criteria of the sample configuration (A); once the initial group of users is selected, the defined procedure (UARP) adapts the inclusion and exclusion criteria based on the potential response to the intervention (B). The intermediate objectives of this procedure (Output 1) will serve as a framework for designing the programming of both the NAO robot and the tablet used as activity mediators throughout the intervention. Following this, data analysis extraction (C1) begins as soon as the intervention sequence is carried out (C2), which will allow for the definition of changes in the user’s instrumental competence (Output 2). (section D, Improvement, in **Figure 3**).

**Figure 4:**
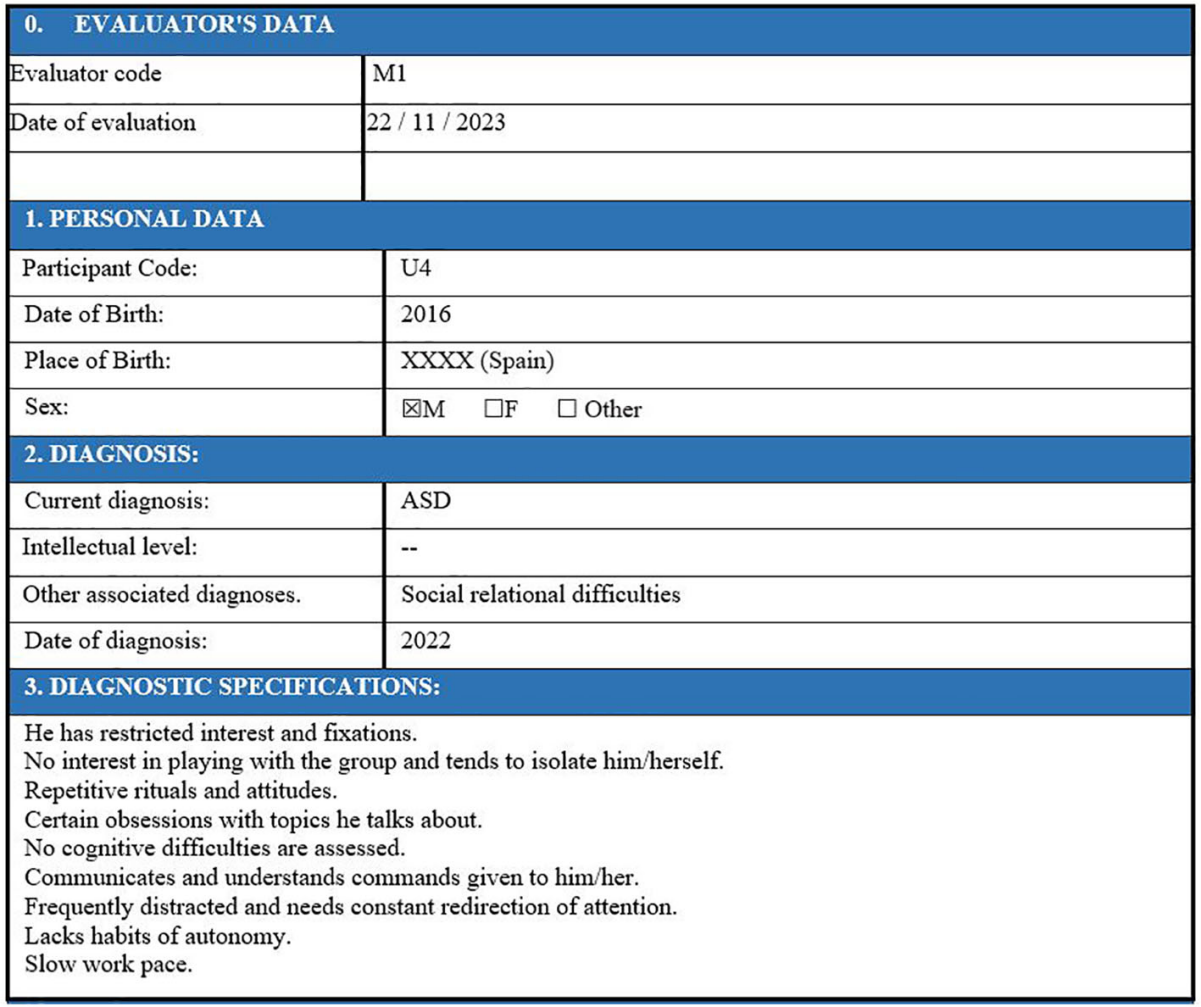
Initial data from the user profile.

**Figure 5:**
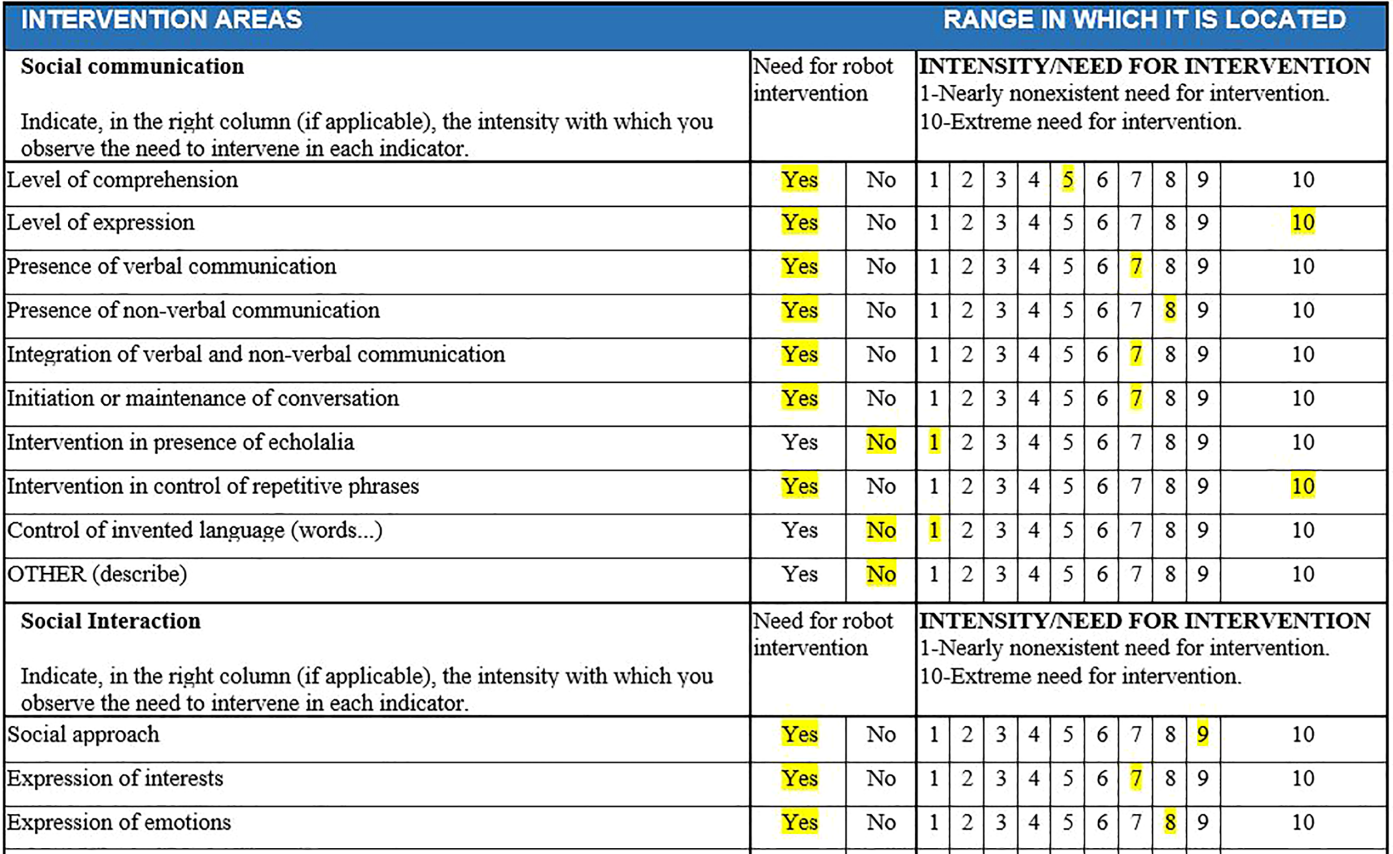
Example of dimension evaluation by evaluators for each child in SIEI. The educator's assessment for a specific user is highlighted in yellow. If "YES" is marked, it is understood that intervention is needed above 3 (usually falling between 5 and 10). If "NO" is marked, it is understood that there is no need for intervention, or it is minimal (1-2).

A total of five experts, all staff members at the educational center and familiar with all students with access to ISIE, participated in the data collection process. The team of specialized educators was selected on the strength of their direct knowledge of each case, based on the analysis variables described in the procedure and the possibility of direct access to each clinical and academic profile. At least two of the five evaluated each case study separately, maintaining anonymity for both the evaluator and the child, as seen in **Figures 4**, **5**.

All the children in the ISIE program (ASD classroom) were assessed by experts, with two to four observation records per child, depending on the number of specialized educators responsible for classroom activities. This point is crucial for future replications: the “experts” evaluating the student separately should be the educators, teachers, tutors, or psychologists who regularly interact with the student and are familiar with their behavior. In the specific case of the study school, students enrolled in the ISIE spend part of their time in regular classes and part of it in the ISIE support unit. The team from this operational unit, who are deeply familiar with the students due to extensive monitoring, are the most suited to determining and classifying the students’ degree of behaviors and aspects that allow them to participate in interventions for their improvement.

For this data collection and analysis process, the project was presented to the Research Ethics Committee of La Salle Campus Barcelona on 15/12/2022 (code 2223-001), who approved this procedure on 15/04/2024 (code CER_URL_2023_2024_009). All families of children in ISIE were informed about the project through informational documents (validated by the ethics committees) and meetings with ISIE professionals, and they all signed consent documents to participate in the project. As indicated, personal data are exclusive to the educational center and ISIE, and anonymized, as verified in **Figures 3**, **4**, they are transferred to the research team for profile weighting and prioritization.

## 3 Results

Observational data were collected and then analyzed. Specific tables were created for each case study, detailing the main results (item and intensity for the three highest values) within each study category (communication, social interaction, and restricted and repetitive behavior).

**Table 1** shows the classified results from the observation of two intervening experts (M1 and M2), who evaluated, for example, user 4 (U4) separately. The procedure involved identifying the highest intensity line in the evaluation (column shaded in green) and the subsequent immediate line by order of aggregate score (shaded in blue). This facilitated not only a judgment of intensity but also a qualitative evaluation pattern based on the highlighted items. For instance, for U4, the profile determined the intensity in item 2 (social communication/level of expression intensity) and item 8 (social communication/need for intervention in control of repetitive phrases) for the social communication dimension; in dimension 2, social interaction, item 5, the need for intervention in shared games was highlighted with greater intensity; finally, in the third dimension, on restrictive and repetitive behavior, the greatest intensity was reached in item 3, the need for intervention in simple motor stereotypies.

**Table 1:** Example (User 4) of data extraction for profile selection.

U4																					
Social communication																					
	1	2	3	4	5	6	7	8	9	10											
M1		10		8				10														
M2		8	7	7	7	7		8														
Social interaction																					
	1	2	3	4	5	6	7	8	9	10	11											
M1	9				10	9	9	10														
M2	10	9	9		10			9														
Restrictive and repetitive behaviors																					
	1	2	3	4	5	6	7	8	9	10	11	12	13	14	15	16	17	18	19	20	21
M1		9	10						10			9		9								
M2			9							9	9											

In green, we find the maximum and common values (identified by the educators) for each dimension. In blue, the secondary value(s) have been marked as relevant in case of a shared identification by the educators (both) of a variable.

The procedure used enabled the observation of the detected level of intervention for each case, the research objective, and the intensity of the need. Thus, the extracted information could also be contrasted by the result obtained from the information dump from each case’s documentation (notes and qualitative data from the center about the needs of each user).

With the analyzed data, a classification of the case studies was created based on the need for intervention and the individual profile’s suitability for the potential benefits derived from communicative and social interaction with the robot. **Table 2** shows the final weighting obtained for all analyzed cases, as well as those discarded due to a lack of information or errors in data capture from the record (NULL).

**Table 2:** Results of the categorization and classification process, ordered by weighting.

User	Mark	Age	
U5	59	7	
U4	57	6	
U8	54	13	
U2	51	6	
U6	51	7	
U1	47	5	
U3	46	6	
U9	46	12	
U7	44	9	
U10	NULL	13	
U11	NULL	3	

The prioritization has been divided into three levels based on the results: Green for high priority; yellow for medium; and orange for low. Red indicates those discarded due to errors in data extraction.

The colors correspond to the resulting prioritization categories. Thus, three profiles suitable for intervention with the robot were extracted, labeled U5, U4, and U8, combining the intensity of the need for intervention with the robot and the profile’s suitability (sensitivity to intervention). Subsequently, the remaining cases were sequenced in decreasing order. Of the 11 studied cases, the first (U5) was discarded due to additional factors provided by the center’s educators and experts, related to a situation of social vulnerability, which could influence subsequent interpretation and contrast. Cases U10 and U11 were also discarded due to the arrangement of unexpected data patterns (homogeneous or incomplete data).

Ultimately, the final extraction of results returned two basic intervention profiles in the study group, prioritized for U4 and U8, aged, as observed at the study time, 6 and 13 years. **Table 3** shows the final information chart for case U4.

**Table 3:** Candidate selection profile.

U4	A. General	
	6-year-old child with a diagnosis from 2022	
	Restricted interests	
	Does not play in groups	
	Tends to isolate	
	Gets distracted	
	Slow work pace	
B. Prioritization and Categorization Indicators		C. Data Dump (Qualitative)
Item 2: Intensity of expression level Item 8: Need for intervention in controlling repetitive phrases		**Social Communication** Expression Level Verbal and Non-verbal Communication Integration of Verbal and Non-verbal Communication Initiation or Maintenance of Conversation Repetitive Phrases Speaks in a Very Low Tone
Item 5: Need for intervention in shared games		**Social Interaction** Expression of Emotions Manifestation of Affection in Their Own Way (Sometimes) Social Interest and Approach Eye Contact Shared Games Theory of Mind Facial Expression
Item 3: Need for intervention in simple motor stereotypies		**Restrictive and Repetitive Behaviors** Alterations in Sensory Processing Changes in Routines Hypo/Hyperreactivity to Sensory Stimuli Repetitive Behaviors Restricted Interests Fascination with Lights Simple Motor Stereotypies Cognitive Inflexibility Always Plays Alone Does Not Play

Case Study U4.

As observed, the extracted profile encompasses a cognitive, social, and behavioral repertoire adapted to the possibilities described in the prior theoretical framework for social intervention with the robot. Concurrently, the conditions derived from the dimension on restrictive and repetitive behavior made it possible to employ devices and sensors such as biosensor device LEDs, cameras, and interaction tablets, and the design of activities (e.g., customizable motivational support for intervention, or design and organization of the physical space for intervention (potentially distracting elements, light, acoustic thresholds, etc.). Simultaneously, the procedure established a baseline that could be applied to other cases (prioritized profile, age, degree of impairment).

## 4 Discussion

The sensitivity to intervention in improving communicative, linguistic, and behavioral competence in autism spectrum disorder is determined by understanding the diversity of profiles. However, under controlled situations regarding the output profile, it is possible to establish an appropriate framework for observing and studying the impact of social uses of robotics. In this sense, studies such as (28) indicate that the robot’s stimulatory simplicity compared to that of humans has a facilitating character regarding shared and sustained social attention mechanisms, enabling the creation of research contexts that make it possible to contrast the interaction effects between both agents.

The contributions of the presented study stem from the need, at the level of current scientific research, to define procedures that shed light on the actual efficacy and behavior of social robotics applied to ASD profiles by developing prior procedures for homogenizing the study sample, given the high variability of symptoms and profiles (age matching, degree of depth, etc.). This mechanism is presented as a clear priority when contrasting not only interaction models between humans and robots but also when determining which therapeutic or intervention procedures are most effective and suitable for each situation (29). This is even more pertinent when, in line with previous works (30), we encounter the problem of the clinical relevance of the intervention, so that control elements of the intervention process can be highlighted (sample selection and homogenization, design, research context, results analysis, impact, and social transformation) to ensure the connection between the effects and the clinical purpose.

The work proposed by Rudovich et al. (22) considers the benefit of personalized intervention adjustment over one-size-fits-all models. However, we identified two conditions to consider concerning our work:

- On one hand, the consideration of the existence of cognitive-behavioral elements that would prioritize the intervention needs of children with ASD, beyond their emotional condition or profile [such as pro-social behaviors, communication and language from a social perspective, or the mentalistic attribution capacity of the autistic student (31)]. This aspect presents the ongoing challenge not only of the robot’s ability to recognize patterns but also of its behavioral adaptation throughout training and intervention. It is in this scenario, linked to language and communication, where the robot’s potential for recognizing qualities still shows room for improvement.
- The second issue highlights the need for generalization from procedures based on a single case and/or session to those that combine multiple sessions and levels of therapeutic intervention over time. Here, the determination of each participant’s cognitive-emotional and linguistic-communicative pattern must be defined to the temporal variable to enable the necessary therapeutic adaptability.

The alignment between means and ends is, therefore, a relevant matter in ASD intervention. Given the diversity of pre-existing patterns, the homogenization of initial profiles, along with the suitability of the intervention object (32), guarantees, in addition to the above, the possibility of applying the effects in other similar situations and contexts, which is significantly relevant when dealing with research processes based on the individual case (at least in the early phases). This fact, coincidentally with previous research (33) constitutes a key position for considering the concept of normality linking the use of social robots in ASD intervention.

Following the prioritization performed, and in a subsequent phase, an interview was conducted with U4’s family to identify aspects that would enable greater customization of each of the 5 interventions with the robot designed. The interview with families is an important part of the process of designing interventions, as it helps identify aspects that will improve the student’s comfort when interacting with the system. Preferred colors, music, animals, characters, cartoons, plants, places, or situations that create a sense of calm (to enhance the interventions), or the opposite (to avoid in any image, sound, color, etc. when we design the interventions), are identified during this interview, and the design is adapted so that the student encounters a comfortable and personalized interface aligned with their preferences, ultimately using examples of interest to them. As we will see below, working with specific numbers, photographs, colors, or emotions is a key aspect that facilitates successful interactions between the student and the robot. In this regard, the interventions focused on:

- Fixed Numbers: U4 remained participative with the robot and consistently expressed a feeling of joy for what they were doing. They expressed emotions in line with what was expected from the activity, constantly looking at the robot and establishing good communication with it, even setting aside interaction with the educator. The only negative point was some initial discomfort with the biometric bracelet, which subsequently disappeared.
- Description of a Photo: Before starting the session, the user expressed their joy at working with the robot again. However, the user communicated less with it, and their responses were simple and repetitive. Compared to the first dynamic, they looked more at the educator, and regarding character imitation, although they liked it, they did not identify all the aspects of the robot.
- Alphanumeric Identification: U4 always followed the same response pattern, seeking continuous interaction with the robot, and demonstrating a good degree of comfort/interaction with it.
- Emotion Recognition: In this session, U4 had many difficulties dragging the images. Conversely, they did identify the emotions, though depending on the image, they found it more or less difficult to identify them.
- Image Identification: In this last session, the user performed the activity very well and showed contentment and eagerness to continue working with the robot.

## 5 Conclusions

In conclusion, the study user demonstrated good adaptation to interaction with the robot and the five dynamics created based on their profile. Processes to improve interaction and usability of work with the robot and tablet were detected, which did not affect U4’s motivation and comfort with the robot and the dynamic itself.

This proposal establishes an alignment framework with current research in a bid to understand the clinical and educational uses of social robotics in the autism spectrum, fomenting the recognition of the existence of specific intervention profiles, based on the analysis of the objective and individual starting conditions, as keys to the valid and reliable observation of the behavior of results and for transfer to new cases, both of a similar pattern and a normal-typical contrast.

The ability to personalize the entry conditions at the behavioral, cognitive, verbal, communicative, and emotional levels for a specific user allows for a more precise determination of the relationship between the outcome achieved and the design of the procedure used with the robot (37). This approach aligns with the current challenges described at the experimental research level, the application level, and the algorithm level (34). In this regard, we find significant current limitations in these types of studies, which present an intriguing avenue for future research. As Artificial Intelligence and ML advance to enable more fluid interaction and adjustment processes (not only for evaluation and diagnosis but also for intervention (35)), we will be able to achieve results based on larger and more generalizable samples. However, at present, it is necessary to have a specific and differentiated body of knowledge to feed these systems, which currently stems from intervention procedures adapted to the interindividual reality (36).

## Data Availability

The raw data supporting the conclusions of this article will be made available by the authors, without undue reservation.
